# Internet-based psychoeducation and support programme for relatives of young people with early psychosis: results of the first German-language intervention

**DOI:** 10.3389/fpsyt.2024.1248526

**Published:** 2024-01-16

**Authors:** Mar Rus-Calafell, Tobias Teismann, Fine Kullmann, Dilara Alatas, Cristina Ballero-Reque, Julia Holewa, Marilena Rüsberg, Martin Brüne, Mercedes Paino, Silvia Schneider

**Affiliations:** ^1^Mental Health Research and Treatment Center, Faculty of Psychology, Ruhr-Universität Bochum, Bochum, Germany; ^2^German Center of Mental Health (DZPG), Partner Site Bochum/Marburg, Bochum, Germany; ^3^LWL University Hospital Bochum, Department of Psychiatry, Psychotherapy and Preventive Medicine, Division of Social Neuropsychiatry and Evolutionary Medicine, Ruhr University Bochum, Bochum, Germany; ^4^Department of Psychology, University of Oviedo, Oviedo, Spain

**Keywords:** early psychosis, family intervention, digital mental health, digitalisation, psychotherapy access

## Abstract

**Background:**

International clinical guidelines recommend Family Interventions (FIs) especially for families of people at early stages of psychosis. The German S3 treatment guideline for schizophrenia gives FIs the highest level of clinical recommendation. However, some family relatives have limited access to these services due to health system constrains. Digital interventions have emerged as a solution to overcome this hindered access to evidence-based family interventions.

**Objective:**

The present pilot study evaluates the feasibility and potential efficacy of the first German moderated online psychoeducation and support programme (ePSP) for relatives of people with early psychosis, with the additional purpose to improve accessibility and reduce waiting times.

**Methods:**

A pre-post study was performed. A brief recruitment period was pre-established (10 weeks) to test potential improvement of regular therapy waiting times in Germany. A total of 25 relatives of people with early psychosis were recruited and received the 12-week moderated online intervention. Assessments were conducted at baseline and at post intervention. Acceptance of the intervention and the user’s experience were also evaluated at post intervention.

**Results:**

Recruitment, retention rates and qualitative data support the feasibility and acceptability of the ePSP. Significant positive effects of the interventions were found on key therapeutic targets, including both primary outcomes (i.e., perceived stress and beliefs about the illness). Twenty-one participants also completed the open-ended questions of the user experience questionnaire, which yielded three main themes: most important modules, difficulties in using the programme and ways to improve ePSP.

**Discussion:**

These results provide preliminary efficacy estimates for a fully powered RCT to investigate superiority (or equipoise) effects of the ePSP in comparison to the routine face-to-face family therapy groups. This future RCT will also allow further exploration of ePSP to improve access to psychological therapy for relatives of young people with psychosis, also in relation to the new ground-breaking Digital Healthcare Act in Germany.

## 1 Introduction

Schizophrenia and related psychotic conditions are severe mental health problems that often have major long-term consequences on individuals’ psychological and physical health, family, relationships, and employment. They are amongst the more debilitating mental health disorders worldwide, with an estimate prevalence of 9.57 per 1,000 (1). Families play a key role in the person’s recovery process, and especially in the early stages of the disease when help is first being sought. Families provide long-term care and continued support for people experiencing psychosis with a high proportion of patients continuing or returning to live with their relatives (2). The caregiver role is often difficult and exerts a significant impact on the carer’s own mental health (3, 4). Care is often associated with high distress and perceived burden, as well as feelings of confusion, guilt, embarrassment, and exhaustion (4, 5). These levels of distress and burden have been found to be greater in carers of people with first episode of psychosis than those at later stages of the course of the disorder (6, 7). Carers’ burden and stress can also lead to a more critical and hostile attitude in caregiving (4), with detrimental effects on the person cared for and their recovery outcomes (8). Caregivers’ negative beliefs, appraisals or perceptions of psychosis play another decisive role in the carer’s functioning and well-being, independently of the affected person’s clinical symptomatology (9). That is, those who appraise the disorder more negatively, in terms of the perceived impact on themselves and the affected individual, are more likely to report greater caregiver burden and stress (10).

Clinical guidelines recommend Family Interventions (FIs) especially for families of people with First Episode of Psychosis (FEP), as early stages of psychosis often occur at a time when many young people are still living at the family home. The German S3 treatment guideline for schizophrenia gives FIs the highest level of recommendation (11). It recommends introducing FIs during a first episode of psychosis, in acute phases as well as in remission stage, especially if the person affected by psychosis lives with or maintains close contact to their family (11). One of the central ingredients of FIs concerns psychoeducation, which comprises information about the disorder, early warning signs and relapse prevention (12). FIs usually include stress reduction and emotional processing techniques, cognitive reappraisal, and structured problem solving (13). Their aim is to enhance carers’ understanding of psychosis, communication skills, coping strategies and problem solving. FIs can be delivered in a single or multiple family setting (14), with the group setting having the added effect of promoting mutual support and simultaneously tackling caregivers’ isolation (15). FIs are always conducted by mental health providers; however, they can differ in their theoretical basis, modality, and length.

Despite the recommendations and seminal work on active ingredients for these interventions, different barriers to professional support for caregivers have been identified, including accessibility, financial pressures, ethnic inequalities in mental health care, and time constraints of health care professionals (6, 12, 16, 17). Caregivers of people with psychosis often face social isolation (18) and stigmatisation, adding more barriers to health care access (12). On a broader level, long waiting times, living in rural residential areas, or scheduling problems have also been identified as challenges to access FIs (at an individual or group level) (13).

Online programmes are emerging as adjunct solutions to these challenges (15, 18, 19). Their advantages include a high flexibility and accessibility for users, whilst considering individual needs and work schedules. This is especially important in the face of carers’ often highly loaded daily routines, but also in the sense of reducing significantly long waiting times for family support. Moreover, such interventions usually do not require a small, limited number of participants (18) as those delivered in a face-to-face group therapy setting. They also allow one to remain anonymous to other participants (if wanted), which may facilitate engagement of relatives into this type of interventions. In the context of psychosis, recent studies have recommended eHealth interventions for family members, as they have been found to be effective at increasing knowledge about the disorder and offering support to relatives (18). Recently, the German Federal Parliament has adopted the Digital Healthcare Act (*Digitale-Versorgung-Gesetz* or DVG) (20). This new law allows individuals covered by statutory health insurance providers to benefit from certain digital health applications which comply with specific criteria. This means that insurers will reimburse payments related to mental health treatments delivered with the support of these digital applications. At the same time, this act promotes the use of telehealth (e.g., video consultations), and better usability of health data for research purposes (20). Amongst the Organisation for Economic Co-operation and Development (OECD) countries, Germany’s DVG represents a first-of-its- kind opportunity for large-scale reimbursement of evidence-based digital health applications.

The present research study is, to our best knowledge, the first study in Germany aiming: (a) to design an online psychoeducation and support programme for families of young people with psychosis; (b) to assess its feasibility (by indicated successful rates of recruitment in a short period of time and retention rates) and acceptability; and (c) to obtain preliminary estimates of its effectiveness on the carer’s perceived distress and psychological well-being.

## 2 Methods

The study was conducted in full accordance with the Declaration of Helsinki, the German data protection act, and the Good Clinical Practice-Guidelines. The study was approved by the local Ethics Committee of the Faculty of Psychology at the Ruhr-Universität Bochum (reference 692/2020).

### 2.1 Design

The present study follows a pre-post within-subjects experimental design to investigate the feasibility and acceptability of a 12-weeks online psychoeducation and support programme for relatives of young people with psychosis.

### 2.2 Participants

Carers of young people with psychosis around Germany were invited to participate in the study. The inclusion criteria were as follows: (1) participants needed to be carers of a relative with a confirmed diagnosis of psychosis [F20-29 in (21)] with onset of the first psychotic episode or first presentation to mental health services in the last 5 years; (2) the participant’s relative must have been aged between 18 and 35 years; (3) participants had to be fluent in German; (4) be able to consent and (5) be able to access the online programme for the following 12 weeks. Exclusion criteria was that the affected relative had a diagnosis of psychosis occurring secondary to a known neurological condition or limited to the context of substance misuse.

### 2.3 Measures

#### 2.3.1 Feasibility

The programme’s feasibility was assessed by recruitment and attrition rates at the end of the programme. The recruitment period was pre-established for 10 weeks (starting from 15th of January 2021), to evaluate recruitment success against average waiting times for psychotherapy in Germany [12.5 weeks for an initial consultation and 23.4 weeks for the first therapy session (22)].

#### 2.3.2 Primary intervention outcomes

*Perceived Stress Scale* [PSS-10; (23, 24)]: This self-reported questionnaire evaluates appraisals to different situations during the last month. This 10-item questionnaire is rated on a 5-point scale ranging from 0 (never) to 4 (very often). The German PSS-10 has high internal consistency (*α* = 0.84) and construct validity (24).

*Illness belief questionnaire* (25), based on items from Broadbent et al. (26) and Lobban et al. (10): Participants were required to read through a series of statements and indicate the degree to which they agreed with the statement on a visual analogue scale anchored from 0 to 100%. Examples: *“How much control do you think your relative has over their illness? How much do you think you are to blame for your relative’s illness?”* Higher scores on each item indicated stronger endorsement of that belief.

#### 2.3.3 Secondary intervention outcomes

*Self-Compassion Scale* [SCS-SF; (27)]: This self-reported questionnaire assesses how individuals act toward themselves in difficult situations using 12 self-report items. On a 5-point scale from 1 (almost never) to 5 (almost always) participants are instructed to indicate how often they behave in the stated manner. A sample item reads as follows: *“I try to be understanding and patient towards those aspects of my personality I do not like.”* The questionnaire was translated to German by two members of the research team and back translated to English by an official translator. Final version was sent to the original authors for final approval. SCS-SF is reported to have good psychometric properties (Cronbach’s α ≥ 0.86 and a near-perfect correlation with the long form SCS r ≥ 0.97 (26)).

*Depression Anxiety and Stress Scale* [DASS-21; (28, 29)]: The DASS-21 comprises 3 subscales with 7 items for each subscale. Participants assess the accuracy of each of the 21 statements on a Likert scale ranging from 0 to 3. Higher values indicate higher levels of psychopathology. The scale proposes a cut-off value of 6 for anxiety and 10 for depression and stress. Internal consistency for nonpsychiatric populations is above 0.75 for all subscales. Reliability can be judged between acceptable and good with a Cronbach’s alpha of 0.88 for depression, 0.80 for anxiety and 0.87 for the stress scale (29).

*Short Form Health 36 Survey* (SF-36) [German version by Bullinger, (30)]: This is a self-reported generic questionnaire of 36 items aiming to assess eight health dimensions: physical functioning (PF), role limitations due to physical health problems (RP), bodily pain (BP), general health perceptions (GH), vitality (VT), social functioning (SF), role limitations due to emotional problems (RE), and general mental health (MH). It does not include a global index but does offer physical and mental component summary scales. Internal consistency was higher than *α* = 0.70 in each dimension (except SF, *α* = 0.45), and test–retest reliability has varied between *r* = 0.58 and 0.99 in all domains (27).

#### 2.3.4 Acceptance and user experience feedback

A user’s experience survey was carried out at post-intervention and used as a reference to explore acceptability of the ePSP. This survey included nine items evaluated on a 5-point Likert scale ranging from 1 (strongly disagree, e.g., *not at all easy*) to 5 (strongly agree, e.g., *very easy*) and three open-ended questions. The Likert items aimed to cover the following topics: simplicity of use, enjoyable, helpful and safety of the programme, usage of the chats and how efficient the programme was in improving participants’ understanding of psychosis. The open-ended questions included were: *“In your opinion, how can the online programme for psychoeducation and support be improved? In your opinion, which are the most important modules of the online programme for psychoeducation and support? In your opinion, what made it difficult to use the online programme for psychoeducation and support?”*

### 2.4 Intervention (ePSP)

The *eHealth Psychoeducation and Support Programme (ePSP):* The programme has been fully developed by licenced psychotherapists and MSc students in clinical psychology at the Mental Health Research and Treatment Centre (Ruhr-Universität Bochum, Germany), and it has been set up as a German online self-learning course on Moodle. Moodle is a free of charge learning platform designed to provide educators, administrators, and learners with a single robust, secure, and integrated system to create personalised learning environments.^1^ The research team worked closely together with the university Moodle programmers on the construction of the ePSP. The content is based on scientific evidence and psychological interventions (mainly cognitive-behavioural therapy, CBT), communication and problem-solving skills learning programmes, self-care interventions (including Mindfulness), and peer-support principles in the context of family interventions for psychosis (3, 4, 13–15).

A round of consultations with clinicians (clinical psychologist and psychiatrist), social workers and service users were held during the development of the programme’s content. These included three online advisory meetings (due to the restrictions of in-person meetings during the COVID pandemic). Feedback from these sessions was integrated into the further elaboration of the online course.

The ePSP comprises five modules: Module 1 (What is Psychosis?) provides psychoeducational information about psychosis (e.g., positive and negative symptoms), what it feels like to experience psychosis, stages, diagnosis and aetiology of psychosis, links to cannabis use, and other related difficulties (e.g., physical health). Module 2 (Treatment & Crisis) covers information on medication, psychotherapy, recovery, crisis, and risk of relapse. Improving communication and understanding the impact of emotions is the main topic of module 3 (Communication & Emotion), whereas module 4 (Self-Compassion) addresses self-acceptance and self-love (i.e., personal strengths, mindfulness, relaxation techniques, sleep hygiene and maintenance of healthy diet). Finally, module 5 (Health Services) provides an orientation on how to navigate the German healthcare system, including early detection, psychiatric (outpatient and inpatients) clinics, psychotherapy praxis, and further contact points.

**Figure 1:**
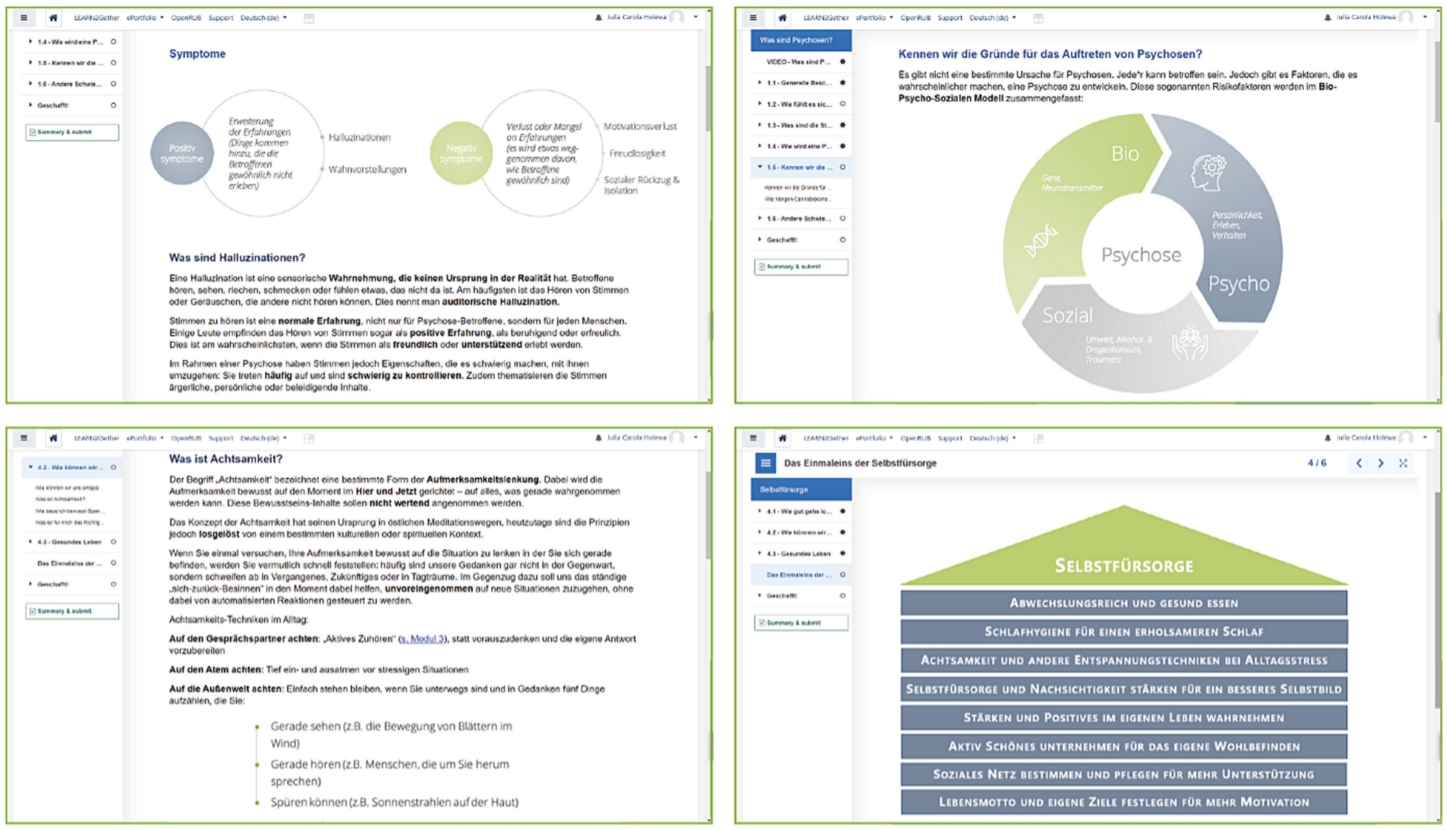
Screenshots from the ePSP interactive website.

The content has mainly been generated in the form of information-based texts, graphics, and audio-visual material (see Figure 1). This material is presented as an “interactive book” to facilitate engagement, monitoring of consulted material and activity completion (percentage of completion is available for each module as well as for the course as a total). To support participants with putting into practise what they learn during the course, all modules (except module 5) included a series of exercises and downloadable material. Crossword puzzles, memory games, and quizzes were integrated to increase participants engagement and allow for playful learning. Each module includes a module-specific chat: this allowed participants to pose questions and to discuss the specific content of each module. Additionally, in a separated chat named ‘*Time for a break’*, participants could post their general contributions or comments. Participants were encouraged to engage in the online chats: sharing their own personal experiences and providing help with difficult situations was highly welcomed. These forums were moderated by the MSc clinical psychology students and supervised by two licensed psychotherapists. These students met weekly with the main supervisor (one of the licensed psychotherapists) and, prior to the start of the study, received a one-semester clinical seminar on etiological models, clinical management, and digital interventions for psychosis. Questions in the forums were answered by the team as well as by participants amongst themselves. Moderators were identifiable by their full names; participants could decide if they wanted their first name or a pseudonym to be displayed.

### 2.5 Procedure

Recruitment started on the 15^th^ of January 2021 and finished on the 19^th^ of March 2021 (with a total duration of 10 weeks). Participants were recruited via printed and digital leaflets distributed amongst peer-supported and relatives’ groups, mental health hospitals, clinics, and private psychotherapy clinics. Additionally, the study was advertised on the Mental Health Research and Treatment Centre (Bochum) website, and social networks such as Facebook and Twitter were used to promote it. Clinical teams and support groups were able to request a study presentation delivered by the research team. Referrals by clinicians and self-referrals were asked to express their interest in the study via the project’s dedicated email address. After referral or self-referral to the study, participants were contacted by researchers to undergo the inclusion and exclusion criteria screening, to check their availability during study period, and to be sent the participant information sheet. Once eligibility was confirmed, the participant attended an online appointment (via secure portal) with one of the research team members. During this appointment, they were asked to sign the consent form, and completed the baseline assessment (approx. 1 h of length).

Afterwards, participants were sent the log-in details to enter the online platform. Each of the participants was assigned to one research team member who was responsible for their guidance throughout the online platform. Every participant received an informative email with a short guideline on how to get registered to the ePSP, and how to navigate it. If participants did not register for the course or no activities on their profile were registered, they received an e-mail reminding them of the programme and offering further assistance if needed.

During the intervention period, the moderators could interact with participants using the ‘*Time for a break*’, the module’s specific chats, and direct private messages. After 2 weeks of participation, moderators sent a “check-in/motivation” email privately to participants. Once the intervention period was finished, the researchers contacted the participants to schedule the online post-intervention assessment appointment. At the end of this assessment, participants were also asked to respond to the user experience survey. The total duration of this appointment was around 1.5 h. Participants were given a 15€ Amazon voucher for their participation in the two assessment sessions. Participants had the right to withdraw from the study at any time.

**Figure 2:**
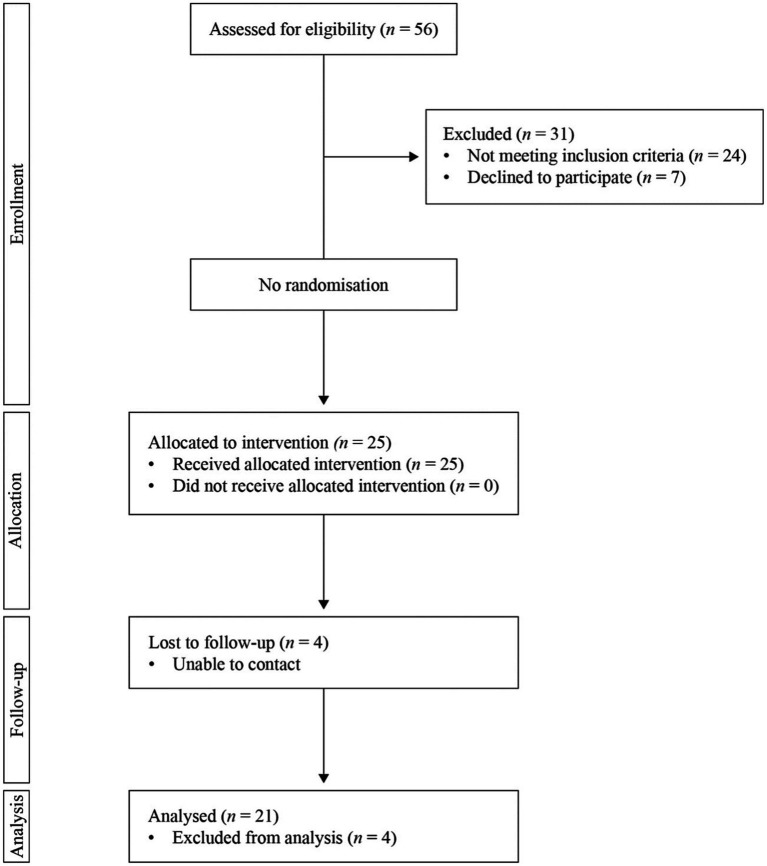
Study flowchart.

Based on the study protocol and pre-established recruitment period (10 weeks, starting from first the advertisement of the study), we aimed to recruit 15–20 participants. A total of 56 people were referred or self-referred to the study (5.6 referrals per week), of whom 25 were eligible and consented to take part in it. Four people did not attend the post-intervention assessment at 12 weeks (16%), although they did complete at least 60% of the online programme (based on available data on activity completion in Moodle). Reasons to not attend the post-intervention assessment are registered in Figure 2.

### 2.6 Statistical analysis

Statistical analyses were performed using RStudio (Version 4.2.0). Descriptive analyses were used to describe the demographic characteristics of the sample. Where the assumption of normality for the difference variable (baseline – post-intervention) was met, repeated measures *t*-test were performed to explore differences between baseline and at post-intervention time points. Otherwise, the non-parametric Wilcoxon Signed Rank-Test was used. Results were considered significant at *p* ≤ 0.05.

The open-ended questions of the user experience survey were analysed using the responsive-reader method used by Sin and colleagues (31). This method aims to minimise the effects of the authors’ views on likely prioritisation of themes whilst maximising the richness (31). This process involves multiple readings by the authors of the given written answers to the open-ended questions, and the subsequent identification of common and repetitive themes. The readings were shared by three of the authors (JH, FK, and MRC) and consisted of three stages: a first read to get familiar with the content, a subsequent stage of identification of the most common and repetitive themes, and a final stage of identification of themes not fitting on the proposed categories. Final consensus about the main themes was reached after these three stages. Direct quotations are given to illustrate the extracted main topics (see 3.4 section below).

## 3 Results

### 3.1 Demographics

Table 1 reports participants’ demographic characteristics at baseline. 88% (*n* = 22) of the participants were native German speakers. All non-native speakers (*n* = 3) reported confidence in reading and understanding a German text. Caregivers were primarily female (92%) and most of them (88%) were a parent of the person experiencing psychosis. Participants were between 23 and 70 years old with an average age of 53.68 years (SD = 11.24).

**Table 1:** Characteristics of carer participants and their cared-for persons (*n* = 25).

	Carer participant	Cared-for person
	*M* (*SD,* age range)	*M* (*SD*, age range)
Age	53.68 (11.24, 23–70)	27.72 (5.6, 18–36)
Age of onset	–	20.52 (4.17, 14–32)
	*n* (%)	*n* (%)
Gender		
Male	2 (8)	16 (64)
Female	23 (92)	9 (36)
Marital status		
Single	1 (4)	–
In relationship	3 (12)	–
Married, cohabiting	17 (68)	–
Divorced	4 (16)	–
Education		
Completed school education, Abitur	6 (24)	–
Completed training	6 (24)	–
Bachelor’s degree	7 (28)	–
Master’s degree/PhD	6 (24)	–
Employment		
Student, vocational training	1 (4)	–
Employed	18 (72)	–
Retired	2 (8)	–
Self-employed	2 (8)	–
Unemployed	2 (8)	–
Monthly net household income		
up to 899€	1 (4)	–
900–2099€	6 (24)	–
2,100–4,099€	7 (28)	–
4,100–5,099€	6 (24)	–
5,100€ and more	5 (20)	–
Relationship with the cared-for person		
Son, daughter	22 (88)	–
Spouse, partner, sibling	2 (8)	–
Cousin, other	1 (4)	–
Clinical diagnosis		
Paranoid schizophrenia	–	18 (72)
Other, not specified	–	7 (28)
Cohabiting		
Yes		17 (68)
No		8 (32)

Participants also gave information about their relatives, who mostly (88%, = 22) were their sons or daughters. Nearly two thirds (64%, = 16) were male, with a mean age of 27.72 years (SD = 5.60, Range 18–35). Most participants’ relative (64%, = 16) had a diagnosis of paranoid schizophrenia. Onset of psychosis had been in average at 20.52 years old (SD = 4.17, Range 14–32).

### 3.2 Feasibility and acceptability

Expected recruitment rate was exceed by 5 participants (total *N* = 25) within the pre-established period (10 weeks). All participants completed at least 60% of the ePSP modules and related activities (based on available data on activity completion in Moodle). However, four participants did not take part in the post-intervention assessment, as they were not reachable by the researchers at that assessment time point (see Figure 2).

Participants’ overall feedback on the programme was positive: They described the programme as “very helpful,” “important” and “valuable.” Relatives were thankful for the opportunity to learn and to be included since usually they feel “left out.” Table 2 depicts caregivers’ responses to the nine Likert quantitative feedback questions at post-intervention. There were no adverse events related to attendance to the programme. Two participants refused to be compensated for their participation in the assessments, as they considered that they were “*compensated enough by having been given the opportunity to attend the programme.”*

**Table 2:** Caregiver evaluation of the acceptance and user experience feedback of the intervention (Likert items) (*N* = 21).

	*M* (*SD*)	Range
Simplicity of use	4.33 (0.86)	2–5
Enjoyment during use	4.48 (0.68)	3–5
Helpfulness of the programme	4.1 (1.14)	2–5
Feeling of safety during use	4.52 (0.75)	2–5
Frequency of chat use	3.19 (1.33)	1–5
Quality of interactions with other participants, *N* = 19	3.68 (1.11)	2–5
Simplicity of interacting with other participants through chat, *N* = 19	3.63 (1.34)	1–5
Helpfulness of the programme regarding the support of one’s relative with psychosis, *N* = 20	3.9 (0.91)	2–5
Efficiency of the programme in improving one’s understanding about psychosis	4.29 (0.72)	3–5

Where different *N* is given, participants were dropped in this analysis due to missing values. Potential range was 1–5 each.

### 3.3 Primary and secondary intervention outcomes

Table 3 depicts pre and post scores of main primary and secondary outcomes of the study.

**Table 3:** Comparison of primary and secondary outcomes at baseline and post-intervention.

		Mean (SD)		Difference		
Measures	Subscales	Baseline (*n* = 25)	Post-intervention (*n* = 21)	*t*	*V*	Value of *p*
PSS						
	Total score	20.43 (10.06)	17.86 (7.81)	1.91		0.035*
	Perceived helplessness	13.48 (6.55)	9.96 (6.45)	2.73		0.006**
	Perceived self-efficacy	9.36 (3.38)	8.32 (4.74)		136.5	0.488
Illness belief questionnaire						
	Consequences (caregiver)	72.32 (22.89)	65.71 (24.41)	0.97		0.172
	Consequences (patient)	86.32 (15.74)	81.62 (15.92)	1.78		0.045*
	Illness control (caregiver)	18.48 (17.43)	23.81 (21.46)	−1.81		0.043*
	Illness control (patient)	33 (21.85)	39.19 (24.18)		55.5	0.058
	Treatment success	78.4 (23.03)	83.33 (15.05)	−1.38		0.092
	Illness cyclical	50.96 (29.01)	59 (27.13)	−0.81		0.215
	Illness understanding	53.52 (28.93)	68.81 (28.37)	−2.87		0.005*
	Coping confidence	49.96 (26.29)	62.43 (23.07)		33	0.007*
	Patient blame	33.4 (32.22)	34.62 (28.74)	0.26		0.400
	Carer blame	20.48 (24.37)	14.71 (13.77)		101	0.256
DASS- 21						
	Depression	6.24 (5.7)	3.29 (3.96)		151	0.002*
	Anxiety	3.71 (3.8)	2.33 (3.01)		105.5	0.005**
	Stress	7.52 (5.68)	5.71 (4.73)		109.5	0.06
SF - 36						
	Physical functioning	90 (11.29)	85.95 (21.25)		61.5	0.728
	Role limitations (emotional problems)	53.97 (40.11)	66.67 (39.44)		22.5	0.314
	Role limitations (physical health)	70.24 (36.76)	73.81 (33.98)		19	0.112
	Energy	45.71 (21.41)	52.86 (20.59)	−2.20		0.020*
	Emotional well-being	54.86 (23.02)	65.14 (19.72)		30	0.005**
	Social functioning	64.88 (30.78)	75 (24.69)	−1.59		0.064
	Pain	64.05 (33.77)	65.29 (35.18)		55.5	0.587
	General health	63.57 (16.59)	64.52 (19.03)	−0.4		0.347
SCS-short form						
	Total score	3.41 (0.7)	3.61 (0.65)	−2.44		0.012*
	Self-kindness	3.07 (0.83)	3.38 (0.74)	−1.78		0.045*
	Self-judgement	2.41 (1.02)	2.17 (0.89)		51.5	0.052
	Common humanity	3.17 (0.78)	3.36 (0.88)		35.5	0.143
	Isolation	3.02 (1.17)	2.60 (1.09)	2.52		0.010**
	Mindfulness	3.55 (0.85)	3.52 (0.93)	0.14		0.556
	Over-identification	2.55 (1.17)	2.5 (1.19)	0.27		0.395

*t*-values are presented when *t*-tests were performed and V-Values when Wilcoxon Signed Rank Tests were conducted. *p < 0.05; **p < 0.01. PSS, Perceived Stress Scale; DASS-21, Depression Anxiety and Stress Scale - 21; SF-36, Short Form Health 36 Survey; SCS, Self-Compassion Scale.

#### 3.3.1 Perceived distress and belief change

Statistically significant reductions in self-reported levels of perceived self-helplessness were observed between pre- and post-intervention assessment (t (20) = 2.73, *p* = 0.006), as well as significant reductions in the total perceived stress score (t (20) = 1.91, *p* = 0.035). Results also indicated a pattern of belief change following intervention in the 10 key beliefs measured. There were significant positive shifts in carers’ beliefs about perceived consequences of the illness for the patient (t (20) = 1.78, *p* = 0.045), the degree of control caregivers have over the illness (t (20) = −1.81, *p* = 0.043), overall understanding of the illness (coherence; t (20) = −2.87, *p* = 0.005). Carers’ confidence in dealing with difficulties showed a significant positive increase (*V* = 33, *p* = 0.007).

#### 3.3.2 Depression, anxiety, and quality of life

Statistically significant improvements in depression (*V* = 151, *p* = 0.002) and anxiety (*V* = 105.5, *p* = 0.005) were observed between pre- and post-intervention assessment. No significant changes were observed in levels of stress (DASS-21 subscale).

Regarding quality of life, results showed an improvement in self-reported levels of energy (t (20) = −2.20, *p* = 0.020) and emotional wellbeing (*V* = 30, *p* = 0.005) between pre- and post-intervention assessment. No significant changes were observed in physical functioning, emotional problems, physical health, social functioning, pain and general health (SF-36 subscales).

#### 3.3.3 Self-compassion

Statistically significant improvements in the total score (t (20) = −2.44, *p* = 0.012), self-kindness (t (20) = −1.78, *p* = 0.045) and isolation (t (20) = 2.52, *p* = 0.01) were observed between pre- and post-intervention assessment. No significant changes were observed in self-judgement, common humanity, mindfulness, and over-identification (SCS subscales).

### 3.4 User experience feedback (open-ended questions)

Twenty of the 21 participants who were available at post-intervention, actively filled in the open-ended questions of the user experience survey.

Three main themes emerged from the thematic analysis of this qualitative feedback.

#### 3.4.1 Most important modules

Twenty relatives stated their opinion on the most important modules. Please note that multiple answers to this question were possible. The first two modules (What is Psychosis?, Treatment & Crisis) covering psychoeducational content were rated as most important by 11 participants each. The modules targeting skills training were considered less important, with nine relatives naming Communication & Emotion and six relatives mentioning Self-Compassion. Being provided with psychoeducational information on the disorder is considered as crucial. One participant noted that for beginners, modules 1 and 2 might be most important, whereas experienced relatives might prefer modules 3 and 4. Several participants (*n* = 4) stated that all modules were equally important. Note that no participant rated Module 5 (Health Services) as essential.

#### 3.4.2 Difficulties in using the programme

We obtained data from 16 relatives on difficulties of the programme. Interestingly, most participants (*n* = 6) answered that using the programme was easy and that they did not experience any difficulties. Some participants (*n* = 5) reported technical difficulties with the Moodle platform, as for example “too many comments” in the chats and needing time to work their way into the platform. Two participants experienced as difficult to read certain “negative posts” from other participants. One participant expressed being *“negatively affected”* by another relative’s comments/self-disclosure of personal experiences with their relative (this participant decided to take a break of several days before re-engaging with the programme).

#### 3.4.3 Ways to improve the programme

Eighteen participants provided ideas and suggestions on how the programme could be improved. These can be summarised in three categories: content, interaction, and technology. Concerning the content: some participants would have appreciated even further information, helpful links, book recommendations and more detailed content on the German health services. One participant wished for more videos and exercises. Interactions were very positively evaluated. Having the opportunity to interact with experts during the programme was much appreciated. One participant would have liked to see a more constructive and solution-oriented exchange between the participants but did not know how to facilitate that. Another participant expressed a similar opinion and provided with a potential solution: *“I would have liked an explanation during the telephone contact before the start of the programme on how you (the research team) envisage using the chat and “Time for a break.” I “dared” to check these chats much too late. After all, the chat is almost as a self-help group itself!”* Another recommendation by a relative was to have users create voluntary profiles including information like their age, relation to the person affected by psychosis and duration of the disorder.

## 4 Discussion

The current study is the first to examine the effects of a German moderated online psychoeducation and support programme (ePSP) for relatives of young people affected by psychosis, including five modules and a moderated online forum. Our results show, firstly, that the intervention is feasible and well accepted by the target group. Secondly, we found preliminary positive effects of the interventions on key targets of the programme, including both primary outcomes (i.e., perceived stress and beliefs about the illness). These benefits were extended to some of the secondary intervention outcomes (depression, anxiety, certain aspects of general quality of life and self-compassion). During the recruitment period, an average of 5.6 referrals/self-referrals were obtained per week. Participants did not wait more than 3 weeks after being assessed to start the programme, which is a clear advantage in terms of accessibility and availability of support. Of the 25 participants recruited, all completed at least 60% of the online programme and 21 (84%) completed the post-intervention assessment. The qualitative feedback was generally positive. However, a few participants reported feeling discouraged by some negative posts from other relatives. Improvements such as including more interactive activities and audio-visual material and having prior more detailed information on how to use interactive chats were reported by some.

The study provides preliminary positive efficacy parameters for the two primary intervention outcomes: perceived stress and illness related cognitions. Improvements in these two domains are consistent to previous studies exploring online psychoeducation and support programmes for relatives of people with psychosis (19, 25, 32). Positive reappraisals by carers can influence coping skills and personal distress. At the same time carer’s appraisals of their relative psychosis influence their interpersonal communication with their relative (19). The ePSP focuses on enhancing positive reappraisals by providing psychoeducation and training about structured problem solving and communication skills. Families’ social isolation and vicarious stigma, and the high value placed on opportunities to share concerns and effectively solve problems with other families (33), suggest that safe and structured online social interaction may provide benefits. This way of interacting with other carers has been suggested to be more cost-effective, promoting engagement and widening dissemination that extends beyond the limitations of the clinic setting (19).

Considering the new German Digital Health Act (DVG), the ePSP would need to undergo further examination in order to be included in the register for digital health applications maintained by the German Federal Institute for Drugs and Medical Devices (Bundesinstitut für Arzneimittel und Medizinprodukte— BfArM). At this point, though, the programme already fulfils 3 of the proposed criteria for it to be eligible for statutory health insurances coverage: the programme is considered a low-risk medical device, its main function is based on digital technology (i.e., Internet-based technology), and it is intended to support treatment in mental health. Further investigations are needed to determine whether insurers and clinicians would routinely prescribe the ePSP to relatives seeking help (remaining criteria).

In light of the results obtained in the present study, including the qualitive feedback from participants, the next iteration of the ePSP programme should include an initial session (either by phone or by video-conference) with a researcher or a guiding video about how the platform works, more interactive material (e.g., videos, games, tasks, downloadable material), and a closer moderation/monitorisation of the effects of self-disclosure of negative experiences from other participants.

The findings of the present study need to be understood in the context of several limitations. Firstly, the current design does not include a comparison to an active/not active control condition. Secondly, most carers were female parents of their adult sons. This reflects a traditional picture of caregiving and has been equally found in previous studies (6, 19, 34). Therefore, generalisability of our findings is not given for other types of caring relationships (e.g., siblings, spouses). External validity could be enhanced by choosing a larger and more heterogenous sample in relation to the caring relationship. Future studies examining the efficacy of the ePSP should also explore the impact of the intervention on the quality of the caregiving relationship, and whether improvements on the illness related cognitions lead to improvements in cognitions held by other members of the household. These and other potential improvements after the intervention should be also explored in the future using in-depth qualitative interviews. Thirdly, recruitment, assessment as well as delivery of the intervention took place during the COVID pandemic. This circumstance led to the temporary closing of many clinical facilities and to the pause or even cancellation of many peer-support groups for relatives (35). The advertisement of the present study in those contexts was impeded, whereby the recruitment strategy had to be shifted almost exclusively from presentation to teams and personal contact to online communication paths. Although we believe that this impeded the familiarity with the research team and potentially hindered higher rates of recruitment, its actual impact on the intervention outcomes is difficult to analyse in the present study. Fourthly, potential benefits of this intervention on the person suffering from psychosis were not monitored. Finally, the platform used to deliver the intervention did not enable exact estimations of the time spent online. This should be a part of future studies, although it is debatable how much usage should be striven for, because being able to use the programme according to one’s needs is an advantage of online interventions.

## Data availability statement

The original contributions presented in the study are included in the article/supplementary materials, further inquiries can be directed to the corresponding author.

## Ethics statement

This study involving humans were approved by Ethics Committee of the Faculty of Psychology at the Ruhr-Universität Bochum (reference 692/2020). This study was conducted in accordance with the local legislation and institutional requirements. The participants provided their written informed consent to participate in this study.

## Author contributions

MR-C and SS conceptualised the study. MR-C, CB-R, JH, MR, MB, and TT designed the intervention (including all written and audio-visual material). CB-R, JH, and MR contributed to continuous moderation of the online intervention. MR-C and TT were responsible of supervising the psychotherapists in charge of moderation of the programme. MP and DA conducted the statistical analysis. MR-C wrote the draft of the manuscript. All authors helped critically revise the article and approved the final manuscript.

## Footnotes

^1^www.moodle.org
